# Association of lumbar vertebral bone marrow and paraspinal muscle fat composition with intervertebral disc degeneration: 3T quantitative MRI findings from the population-based KORA study

**DOI:** 10.1007/s00330-022-09140-4

**Published:** 2022-10-14

**Authors:** Matthias Jung, Susanne Rospleszcz, Maximilian T. Löffler, Sven S. Walter, Elke Maurer, Pia M. Jungmann, Annette Peters, Johanna Nattenmüller, Christopher L. Schlett, Fabian Bamberg, Lena S. Kiefer, Thierno D. Diallo

**Affiliations:** 1 Department of Diagnostic and Interventional Radiology, University Medical Center Freiburg, Faculty of Medicine, University of Freiburg, Hugstetter Strasse 55, 79106 Freiburg, Germany; 2 Institute of Epidemiology, Helmholtz Zentrum München, German Research Center for Environmental Health, Neuherberg, Oberschleißheim, Germany; 3 Department of Epidemiology, Institute for Medical Information Processing, Biometry and Epidemiology, Ludwig-Maximilians-University München, Munich, Germany; 4 Department of Diagnostic and Interventional Neuroradiology, School of Medicine, Klinikum Rechts Der Isar, Technical University of Munich, Munich, Germany; 5 Department of Diagnostic and Interventional Radiology, Eberhard Karls University of Tuebingen, Tuebingen, Germany; 6 Division of Musculoskeletal Radiology, Department of Radiology, NYU Grossman School of Medicine, 660 1st Ave, New York, NY 10016 USA; 7 Department of Trauma and Reconstructive Surgery, BG Unfallklinik, Schnarrenbergstraße 95, 72070 Tuebingen, Germany

**Keywords:** Intervertebral disc degeneration, Skeletal muscle, Bone marrow, Magnetic resonance imaging, Biomarker

## Abstract

**Objective:**

To assess the association of lumbar bone marrow adipose tissue fat fraction (BMAT-FF) and paraspinal muscle proton density fat fraction (PDFF) and their interplay with intervertebral disc degeneration (IVDD).

**Methods:**

In this retrospective cross-sectional study based on a prospective population-based cohort, BMAT-FF and PDFF of asymptomatic individuals were calculated based on 3T-MRI dual-echo and multi-echo Dixon VIBE sequences. IVDD was assessed at motion segments L1 to L5 and dichotomized based on Pfirrmann grade ≥ 4 and/or presence of other severe degenerative changes or spinal abnormalities at least at one segment. Pearson’s correlation coefficients were calculated for BMAT-FF and PDFF. Univariable and multivariable logistic regression models for IVDD were calculated.

**Results:**

Among 335 participants (mean age: 56.2 ± 9.0 years, 43.3% female), the average BMI was 27.7 ± 4.5 kg/m^2^ and the prevalence of IVDD was high (69.9%). BMAT-FF and PDFF were significantly correlated (*r* = 0.31–0.34; *p* < 0.001). The risk for IVDD increased with higher PDFF (OR = 1.45; CI 1.03, 2.04) and BMAT-FF (OR = 1.56; CI 1.16, 2.11). Pairwise combinations of PDFF and BMAT-FF quartiles revealed a lower risk for IVDD in individuals in the lowest BMAT-FF and PDFF quartile (OR = 0.21; CI 0.1, 0.48). Individuals in the highest BMAT-FF and PDFF quartile showed an increased risk for IVDD (OR = 5.12; CI 1.17, 22.34)

**Conclusion:**

Lumbar BMAT-FF and paraspinal muscle PDFF are correlated and represent both independent and additive risk factors for IVDD. Quantitative MRI measurements of paraspinal myosteatosis and vertebral bone marrow fatty infiltration may serve as imaging biomarkers to assess the individual risk for IVDD.

**Key Points:**

*• Fat composition of the lumbar vertebral bone marrow is positively correlated with paraspinal skeletal muscle fat*.

*• Higher fat-fractions of lumbar vertebral bone marrow and paraspinal muscle are both independent as well as additive risk factors for intervertebral disc degeneration*.

*• Quantitative magnetic resonance imaging measurements of bone marrow and paraspinal muscle may serve as imaging biomarkers for intervertebral disc degeneration*.

## Introduction

Intervertebral disc degeneration (IVDD) is a common condition, characterized by the degradation of extracellular matrix and the loss of hydrophilic matrix molecules in the nucleus pulposus [1, 2]. Structural and biomechanical alterations due to the loss of disc height lead to changes in load distributions across the spine, eventually causing degeneration, segmental instability, and facet joint arthropathy [3, 4].

Fatty infiltration of skeletal muscle, i.e., myosteatosis, is associated with muscle quality and skeletal muscle deterioration [5]. Muscle deterioration may have a significant impact on the onset, progression, and severity of osteoarthritis (OA) [6, 7]. Moreover, previous studies reported an association of paraspinal myosteatosis and IVDD, raising the possibility of a “whole-organ pathology” [8–10].

Vertebral bone marrow adipose tissue (BMAT) plays an important role in bone health. Changes in its composition have been associated with various metabolic diseases, such as type 2 diabetes mellitus (T2DM) or osteoporosis [11, 12]. The avascular intervertebral disc is supplied with nutrients via microvessels of the vertebral bodies. Since the conversion of hematopoietic bone marrow into bone marrow fat is associated with a decrease in blood flow and thus nutrient supply to the intervertebral discs, the amount of BMAT may play a role in the development of IVDD [13–15].

Chemical shift encoding-based water-fat magnetic resonance imaging (MRI) is a non-invasive method that provides quantitative information about the biochemical water-fat composition of bone marrow and skeletal muscle in vivo [16, 17]. It allows measurements of the vertebral BMAT fat fraction (BMAT-FF) and the paraspinal muscle proton density fat fraction (PDFF) [18, 19].

Despite spatial and possible functional relationships of paraspinal PDFF and vertebral BMAT-FF, their interactions and potential role in the development of IVDD remain uncertain. Therefore, we systematically analyzed the correlation between paraspinal PDFF and vertebral BMAT-FF and investigated their associations with IVDD in individuals from the general population. We hypothesized that paraspinal PDFF is correlated with vertebral BMAT-FF and that both high paraspinal PDFF and high vertebral BMAT-FF are associated with IVDD.

## Materials and methods

### Study population and ethics approval

**Fig. 1:**
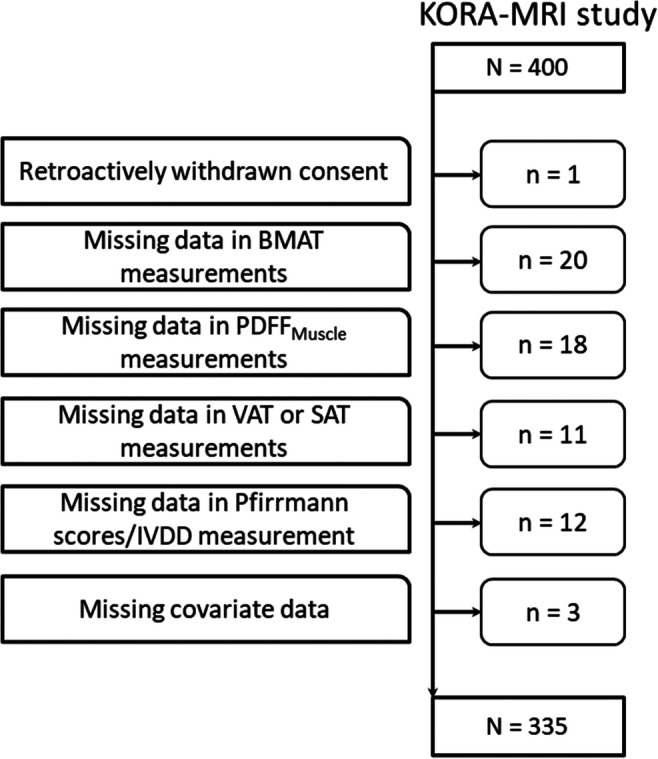
Flow diagram of the study population

The study was designed as a retrospective cross-sectional analysis based on a prospective cohort from the “Cooperative Health Research in the Region of Augsburg” (KORA) [20, 21]. Subjects for this analysis were derived from the KORA-FF4 study (06/2013–09/2014, *N* = 2279), the second follow-up study of the KORA S4 cohort (baseline survey 1999–2001, *N* = 4216) which included a large sample of individuals aged between 25 and 74 years from the general population. From the KORA FF4-study, a total of 400 eligible subjects underwent whole-body MRI according to previously described inclusion and exclusion criteria [21]. For the present retrospective analysis, 65 individuals (16%) were excluded due to insufficient image quality, incomplete MRI data, missing values in any of the covariates, or withdrawn consent to the study (Fig. 1).

The study was IRB approved by the ethics committee of the Bavarian Chamber of Physicians, Munich, Germany, and the local institutional review board of the Ludwig-Maximilians University Munich, Germany. Written informed consent was obtained from all participants.

### MR imaging protocol and image analysis

MRI examinations were performed in a supine position on a 3 Tesla Magnetom Skyra (Siemens Healthineers) using an 18-channel body surface coil and a table-mounted spine matrix coil.

#### Proton density fat fraction (PDFF) of paraspinal muscle

The degree of myosteatosis of lumbar paraspinal muscles was determined as the mean proton density fat fraction (PDFF). PDFF maps were automatically calculated as DICOM files using the software MR LiverLab (Version VD13, Siemens Healthineers) from a multi-echo Dixon VIBE sequence covering the upper abdomen (slice thickness 4 mm, voxel size 1.8 × 1.8 mm, field-of-view 393 × 450 mm, matrix 256 × 179, TR 8.90, TEs 1.23, 2.46, 3.69, 4.92, 6.15, 7.38 ms, flip angle 4°).

Skeletal muscle was segmented in PDFF maps on axial slices at the level of the third lumbar vertebra, as this height has been determined previously as a good surrogate for lumbar and overall skeletal muscle composition [22]. Details of the segmentation and post-processing procedure have earlier been described [19]. In brief, muscle compartments of the quadratus lumborum muscle (PDFF_quadratus_) and the autochthonous back muscles (PDFF_autochthonous_) were manually segmented according to standardized, anatomical landmarks using dedicated software (MITK V2015.5.2, German Cancer Research Center). PDFF of the quadratus lumborum muscle and the autochthonous back muscles were also averaged as PDFF_muscle_. Segmentation was performed by two individual observers fully blinded to clinicopathological data.

#### Bone marrow adipose tissue fat fraction (BMAT-FF) of lumbar vertebrae

Fatty infiltration in cancellous bone of lumbar vertebrae was assessed as bone marrow adipose tissue fat fraction (BMAT-FF). Water and fat selective images in coronally acquired dual-echo Dixon VIBE sequence (slice thickness 1.7 mm, voxel size 1.7 × 1.7 mm, field-of-view 488 × 716 mm, matrix 256 × 256, TR 4.06 ms, TEs 1.26 and 2.49 ms, flip angle 9°) were used to calculate

$$ BMAT\_ FF=\frac{mean\ intensity\  fat\  image}{mean\ intensity\  fat\  image+ mean\ intensity\ water\ image}. $$

Mean intensity values were manually extracted from Dixon images using dedicated software (OsiriX 7.0, Pixmeo SARL) as previously described [18]. Intensity values in a single coronal image passing through the middle anterior-posterior diameter of L1 and L2 were extracted. Therefore, masks of cancellous bone were delineated on the fat image and copied to the water image. Extracted intensity values of masks of L1 and L2 were averaged (BMAT-FF_L1/L2_).

#### Intervertebral disc degeneration (IVDD)

Intervertebral discs were assessed on T2-weighted single-shot fast spin-echo sequences (TR 1000 ms, TE 91 ms, flip angle 131°, slice thickness 5 mm). IVDD of motion segments L1 to L5 were evaluated using the Pfirrmann grading system [23]. Therein, disc structure, distinction between nucleus and annulus, signal intensity, and height of intervertebral disc are analyzed [23]. Grading was defined as follows: grade I: intervertebral disc as homogenous white structure; grade 2: intervertebral disc as inhomogeneous structure with or without horizontal bands; grade 3: annulus fibrosus and nucleus pulposus clearly distinct; grade 4: subtotal collapse of the intervertebral disc; grade 5: total collapse of the intervertebral disc [24].

IVDD was dichotomized into presence/absence of severe degeneration based on a Pfirrmann grade ≥ 4 and/or disc bulging/herniation at any of the levels, presence of scoliosis, diagnosis of pathological vertebrae, or prior intervertebral disc surgery. Details on the reader characteristics, intra- and inter-reader agreements, and reliability measures have previously been described [25].

#### Subcutaneous and visceral adipose tissue (SAT and VAT)

Trunk adipose tissue compartments were segmented and quantified in fat-selective images by a semi-automated algorithm based on fuzzy-clustering [26, 27]. Fat-selective axial tomograms (slice thickness 5 mm, zero-gap) were calculated based on a three-dimensional dual-echo Dixon VIBE sequence (slice thickness 1.7 mm, voxel size 1.7 × 1.7 mm, field-of-view 488 × 716 mm, matrix 256 × 256, TR 4.06, TE 1.26 and 2.49, flip angle 9°). Volumes of SAT and VAT were quantified from the cardiac apex to the femoral head and from the diaphragm to the femoral head, respectively [21, 28].

#### Body mass index (BMI) and waist circumference

The body mass index (BMI) was calculated as body weight in kg divided by body height squared in m^2^, with weight and height both being measured at the study center. Waist circumference was measured at the smallest abdominal circumference or, in individuals with obesity, in the midpoint of the lowest rib and the upper margin of the iliac crest.

#### Physical activity

Physical activity of study participants was assessed by a standardized questionnaire. A dichotomous variable was calculated with (A) physically active participants (regular physical activity ≥ 2 h/week or ca. 1 h/week) or (B) physically inactive participants (irregular physical activity < 1 h/week, almost no and no physical activity) [24].

### Statistical analysis

Baseline characteristics of the study population are presented as mean (±) standard deviation or median and interquartile range (IQR) for continuous variables and absolute counts with percentages for categorical variables, respectively. Correlations between PDFF_quadratus_ or PDFF_autochthonous_ and vertebral BMAT-FF_L1/L2_ were assessed by Pearson’s correlation coefficients. Muscle PDFF and BMAT-FF according to maximum Pfirrmann grade and the number of severely degenerated lumbar spine levels was assessed graphically by boxplots and quantified by one-way ANOVA. The associations between the presence of IVDD as a dichotomous outcome and different risk factors were assessed by logistic regression models, adjusted for age, sex, BMI, and physical activity. To assess the independent effects of PDFF and BMAT-FF on IVDD, logistic regression models were calculated including both PDFF and BMAT-FF as predictor variables. To assess the combined effects of PDFF and BMAT-FF, sex-specific quartiles were calculated and individuals were grouped as follows: (i) both PDFF and BMAT-FF in the upper quartile, (ii) both PDFF and BMAT-FF above the median, (iii) PDFF and BMAT-FF in the lowest quartile. An adjusted logistic regression model with the respective combination as a predictor was then calculated. For all models, continuous measures were standardized (by subtracting the mean and dividing by standard deviation) before modeling. Results are presented as odds ratios (OR) with corresponding 95% confidence intervals (CI). Statistical significance was indicated by *p*-values ≤ 0.05. Statistical analysis was performed using R V4.0.3 (R Core Team, www.r-project.org, 2020). Details regarding the intra- and inter-reader reproducibility analysis and statistical model fitting for BMAT-FF and PDFF measurements have previously been described [18, 19].

## Results

### Study population

The final sample comprised 335 participants (145 women and 190 men) with a mean age of 56.2 ± 9.0 years and a mean BMI of 27.7 ± 4.5 kg/m^2^ (Table 1). Regarding physical activity, 30.4% (*n* = 102) performed exercise of 2 h regularly or more per week, 31.0% (*n* = 104) about 1 h regularly per week, 15.5% (*n *= 52) about 1 h irregularly per week, and 23.0% (*n* = 77) reported no or nearly no exercise.

**Table 1:** Demographics and characteristics of the study sample

	**All individuals**	**IVDD**	**No IVDD**	***p***
	*N* = 335	*N* = 234	*N* = 101	
Age (years)	56.2 ± 9.0	57.9 ± 8.9	52.2 ± 8.1	< 0.001
Male sex	190 (56.7%)	139 (59.4%)	51 (50.5%)	0.165
Postmenopause	98 (29.3%)	71 (30.3%)	27 (26.7%)	0.0102
Body composition				
BMI (kg/m^2^)	27.7 ± 4.5	27.9 ± 4.4	27.3 ± 4.6	0.29
Waist circumference (cm)	97.4 ± 13.4	98.2 ± 13.0	95.7 ± 14.2	0.108
VAT (cm^2^)	146.9 ± 86.3	153.3 ± 83.8	132.1 ± 90.6	0.039
SAT (cm^2^)	7.9 ± 3.3	271.1 ± 106.3	280.3 ± 113.9	0.479
BMAT-FFL1 (%)	52.4 ± 10.6	54.4 ± 10.3	47.8 ± 10.0	< 0.001
BMAT-FFL2 (%)	55.9 ± 10.7	58.0 ± 9.9	50.9 ± 10.7	< 0.001
BMAT-FFL1/L2 (%)	54.1 ± 10.5	56.2 ± 9.9	49.3 ± 10.2	< 0.001
PDFFauthochtonous (%)	17.2 ± 7.9	18.2 ± 8.3	14.9 ± 6.6	0.001
PDFFquadratus (%)	6.7 ± 3.7	7.2 ± 4.0	5.5 ± 2.6	< 0.001
PDFFmuscle (%)	11.6 ± 4.8	12.0 ± 4.7	10.6 ± 4.7	0.013
Physical activity				0.025
Exercise regularly > 2 h/week	102 (30.4%)	69 (29.5%)	33 (32.7%)	
Exercise regularly ca.1 h/week	104 (31.0%)	65 (27.8%)	39 (38.6%)	
Exercise irregularly ca.1 h/week	52 (15.5%)	36 (15.4%)	16 (15.8%)	
Almost no/no physical activity	77 (23.0%)	64 (27.4%)	13 (12.9%)	

Data are presented as means ± SD for continuous variables and counts and percentages for categorical variables. *p*-values from t-test and χ^2^ test, respectively

*BMI* body mass index, *VAT* visceral adipose tissue, *SAT* subcutaneous adipose tissue

### Muscle composition and bone marrow adipose tissue fat fraction

PDFF_autochthonous_ was significantly higher in individuals with IVDD (18.2 ± 8.3 %) compared to those without IVDD (14.9 ± 6.6 %, *p* = 0.001, Table 1). A similar pattern was observed for PDFF_quadratus_ (7.2 ± 4.0% vs 5.5 ± 2.6%, *p* < 0.001) and BMAT-FF_L1/L2_ (56.2 ± 9.9 % vs 49.3 ± 10.2%, *p* < 0.001)

**Fig. 2:**
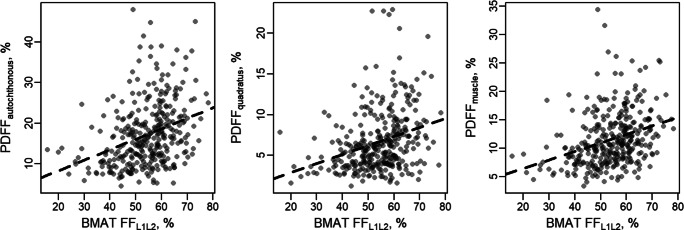
Scatter plots of the correlation between BMAT-FF_L1/L2_ and the various PDFF measurements. BMAT-FF, bone marrow adipose tissue fat-fraction; PDFF, proton-density fat-fraction

There were moderate, but statistically significant correlations between BMAT-FF and PDFF (*r* = 0.31 to 0.34; all *p* < 0.001; Fig. 2). After stratification for the presence of IVDD, all correlations between BMAT-FF and PDFF remained statistically significant (all *p* < 0.001).

### PDFF and BMAT-FF according to Pfirrmann grade and multilevel disc degeneration

**Fig. 3:**
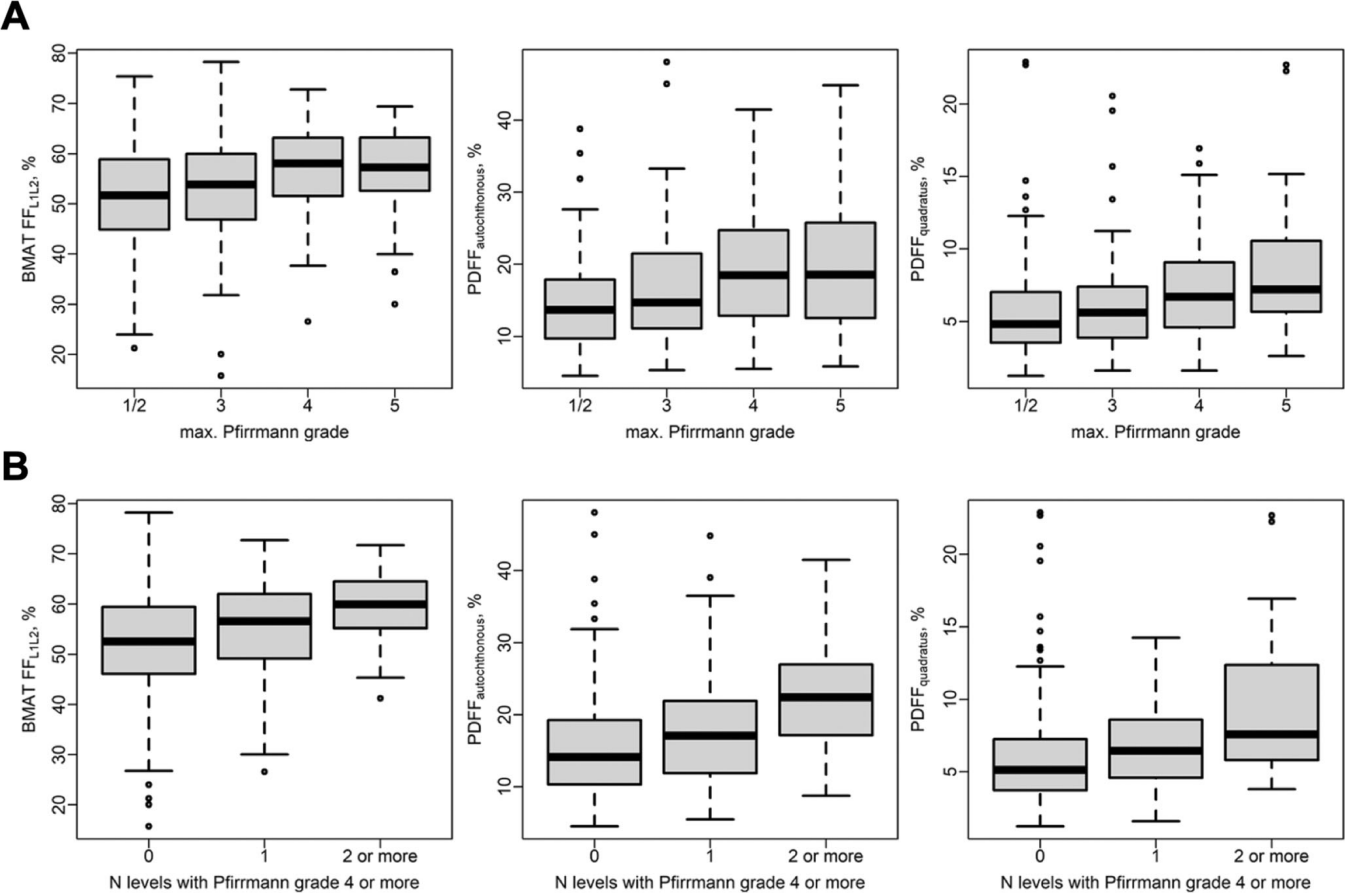
BMAT-FF and PDFF according to Pfirrmann grade and multilevel disc degeneration. BMAT-FF and PDFF according to maximum Pfirrmann grade of motion segments L1 to L5 (**A**) and the number of severely degenerated (Pfirrmann ≥ 4) lumbar spine levels (**B**). BMAT-FF, bone marrow adipose tissue fat-fraction; PDFF, proton-density fat-fraction

Muscle PDFF and BMAT-FF increased according to maximum Pfirrmann grade of motion segments L1 to L5 (all *p* < 0.001; Fig. 3a), however plateaued at Pfirrmann grade 4 where fatty infiltration was comparable to Pfirrmann grade 5. Furthermore, we observed a gradual increase in muscle PDFF and BMAT-FF according to the number of severely degenerated lumbar spine levels, as defined by Pfirrmann grade 4 or higher (all *p* < 0.001; Fig. 3b).

### Association of PDFF and BMAT-FF with IVDD

The overall prevalence of IVDD in our cohort was 69.9% (Table 2). In logistic regression analysis adjusted for age, sex, BMI, and physical activity, PDFF_quadratus_ was associated with higher risk of IVDD (OR = 1.45; CI 1.03, 2.04; *p* = 0.035) as well as BMAT-FF_L1/2_ (OR = 1.56; CI 1.16, 2.11; *p* = 0.003; Table 3). In contrast, PDFF_autochthonous_, PDFF_muscle_, waist circumference, BMI, VAT, or SAT showed no significant association with IVDD (Table 3).

**Table 2:** Frequency of spinal pathologies and IVDD of the study sample

	All individuals
	*N* = 335
Distribution of Pfirrmann scores	
Pfirmann score 1/2 L1/L2–L5/S1	108 (32.2%)
Pfirmann score 3 L1/L2–L5/S1	94 (28.1%)
Pfirmann score 4 L1/L2–L5/S1	88 (26.3%)
Pfirmann score 5 L1/L2–L5/S1	45 (13.4%)
Disc bulging or herniation level L1/L2 to L5/S1	162 (48.4%)
Presence of scoliosis	54 (16.1%)
Pathological vertebrae	6 (1.8%)
Prior vertebral surgery	< 3
IVDD	234 (69.9%)

IVDD was dichotomized into presence/absence of severe degeneration based on a Pfirrmann grade ≥ 4 and/or disc bulging/herniation at any of the levels, presence of scoliosis, diagnosis of pathological vertebrae, or prior intervertebral disc surgery

**Table 3:** Association of different risk factors with outcome IVDD

Risk factor	Adjustment	OR	(95%-CI)	*p*-value
Waist circumference	Crude	1.21	[0.96, 1.54]	0.108
	Adjusted	0.82	[0.42, 1.59]	0.553
BMI	Crude	1.14	[0.9, 1.45]	0.289
	Adjusted	0.96	[0.74, 1.24]	0.769
VAT	Crude	1.29	[1.01, 1.66]	**0.040**
	Adjusted	0.81	[0.55, 1.19]	0.287
SAT	Crude	0.92	[0.73, 1.16]	0.479
	Adjusted	0.65	[0.4, 1.07]	0.090
Physical activity (regular 1 h or > 2 h/week)	Crude	0.74	[0.58, 0.95]	**0.016**
	Adjusted	0.74	[0.57, 0.96]	**0.023**
PDFFautochthonous	Crude	1.61	[1.22, 2.11]	**0.001**
	Adjusted	1.25	[0.86, 1.81]	0.236
PDFFquadratus	Crude	1.84	[1.34, 2.52]	**< 0.001**
	Adjusted	1.45	[1.03, 2.04]	**0.035**
PDFFmuscle	Crude	1.39	[1.07, 1.8]	**0.015**
	Adjusted	1.03	[0.74, 1.43]	0.868
BMAT-FFL1/L2	Crude	1.99	[1.54, 2.58]	**< 0.001**
	Adjusted	1.56	[1.16, 2.11]	**0.003**

Results from logistic regression models with outcome IVDD. Crude: no adjustment, Adjusted: adjusted for age, sex, BMI, and physical activity

*IVDD* intervertebral disc degeneration, *OR* odds ratio, *CI* confidence interval, *BMI* body mass index, *VAT* visceral adipose tissue, *SAT* subcutaneous adipose tissue, *BMAT-FF* bone marrow adipose tissue fat-fraction, *PDFF* proton-density fat fraction

### Independent and combined associations of BMAT-FF and PDFF

In an adjusted multivariable logistic regression model including both PDFF and BMAT-FF variables, odds ratios for PDFF and BMAT-FF only slightly attenuated compared to those in univariable logistic regression (Table 4) and estimates for BMAT_FF remained statistically significant.

**Table 4:** Association of pairwise combinations of PDFF and BMAT variables with outcome IVDD

	OR	(95%-CI)	*p*-value
PDFFauthochtonous	1.19	[0.82, 1.73]	0.350
BMAT-FFL1/L2	1.55	[1.15, 2.08]	**0.004**
PDFFquadratus	1.37	[0.97, 1.94]	0.076
BMAT-FFL1/L2	1.52	[1.12, 2.05]	**0.007**

Results from logistic regression models with outcome IVDD. Predictors were PDFF_authochtonous_ and BMAT-FF_L1/L2_ (upper row) or PDFF_quadratus_ and BMAT-FF_L1/L2_ (lower row)

Adjusted for age, sex, BMI, and physical activity. *BMAT-FF* bone marrow adipose tissue fat-fraction, *PDFF* proton-density fat fraction

**Fig. 4:**
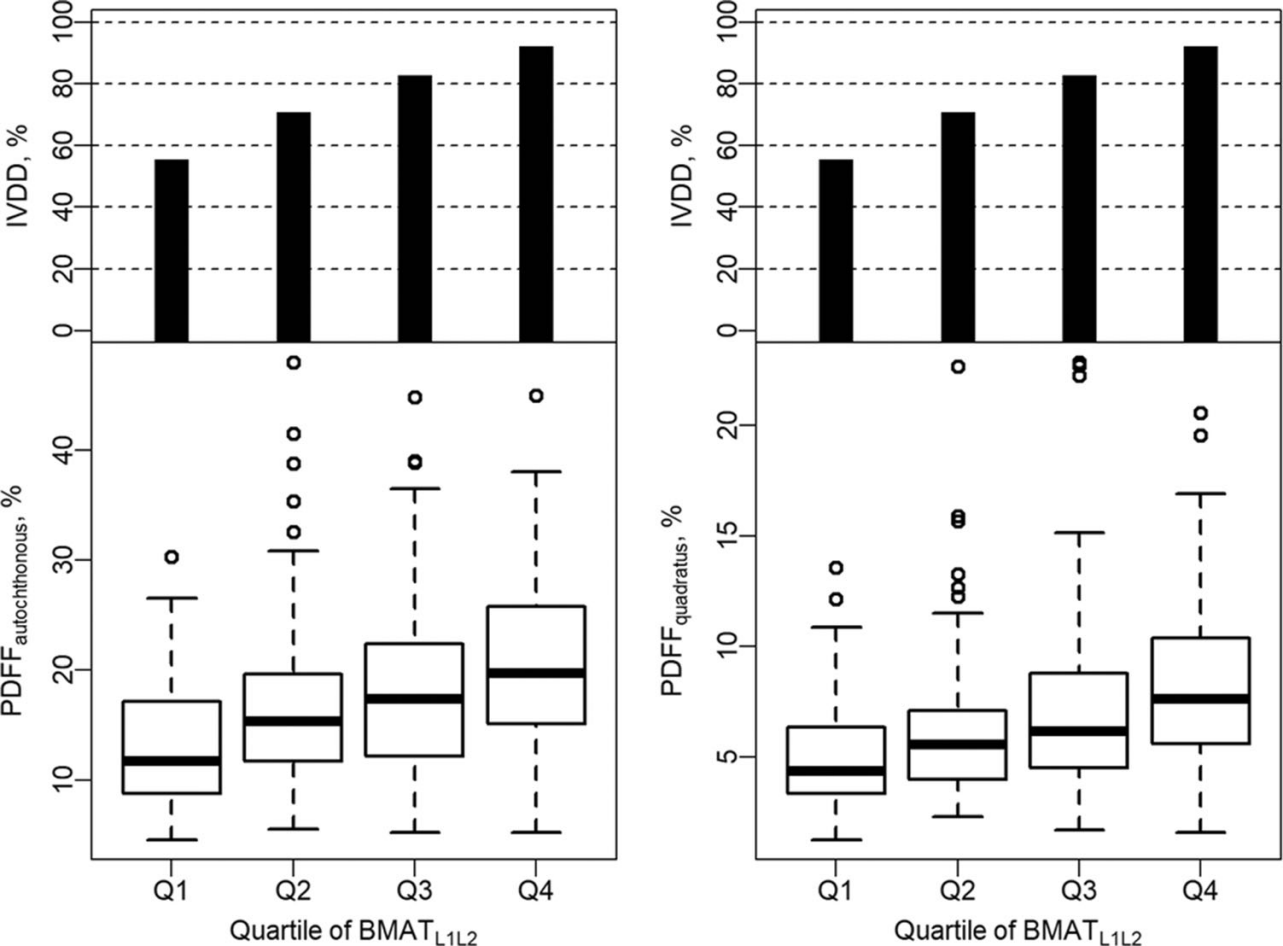
Distribution of PDFF and frequency of IVDD in BMAT quartiles. Stepwise increase in PDFF and the frequency of IVDD for each BMAT-FF_L1/2_ quartile

**Fig. 5:**
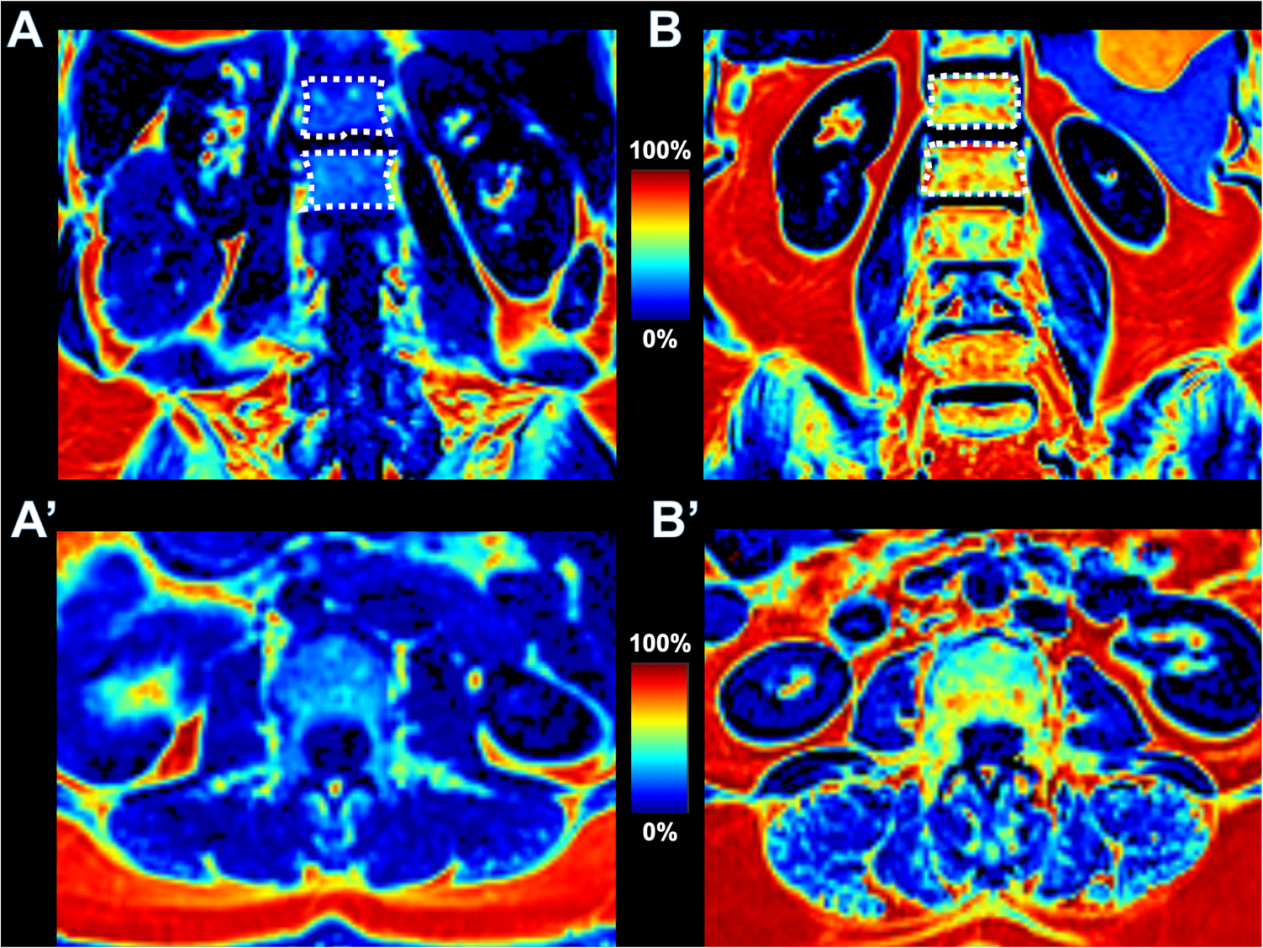
Visualization of BMAT-FF and PDFF in an individual with and without IVDD. Coronal BMAT-FF-maps of vertebral bone marrow (**A**, **B**) and axial PDFF maps at L3 (A’, B’). (A, A’) A female subject without IVDD (Pfirrmann grade 2). Quantitative analyses revealed low fat-fractions in the vertebral bone marrow (A, BMAT-FF_L1/L2_ 27.7%) and in the paraspinal muscles (A’, PDFF 7.5%). (B, B’) A female subject with IVDD (Pfirrmann grade 5). Quantitative maps show increased fat-fractions in the vertebral bone marrow (B, BMAT-FF_L1/L2_ 65.3%) and in the paravertebral muscles (B’, PDFF 19.4%). BMAT-FF, bone marrow adipose tissue fat-fraction; PDFF, proton-density fat-fraction. Blue color indicates low, and red color indicates high fat-fractions. Dotted line indicates region of interest

There was a stepwise increase in mean PDFF_autochthonous_ and PDFF_quadratus_ for each BMAT-FF_L1/2_ quartile (Fig. 4). Further, a stepwise increase in the frequency of IVDD was found across quartiles (Fig. 4). The analysis of quartile combinations of BMAT-FF and PDFF yielded an association of the lowest quartile with a lower risk for IVDD (OR = 0.21; CI 0.1, 0.48; *p* = < 0.001; Table 5; Fig. 5). Moreover, there was a statistically significant higher risk for IVDD for the combination of the highest quartile of BMAT-FF_L1/L2_ with the highest quartile of PDFF_quadratus_ (OR = 5.12, CI 1.17, 22.34; *p* = 0.030; Table 5, Fig. 5). Considering the pairwise combinations of the BMAT-FF_L1/L2_ quartiles and the quartiles of PDFF_autochthonous_, the combination of BMAT-FF_L1/L2_ > median and PDFF_authochthon_ > median was significantly associated with a higher relative risk of IVDD (OR = 2.37, CI 1.17, 4.79; *p* = 0.016; Table 5).

**Table 5:** Association of pairwise quartile combinations of PDFF and BMAT variables with outcome IVDD

Quartile combination: both PDFF and BMAT…		Adjustment	OR	(95%-CI)	*p*-value
PDFFauthochtonous	…in the upper quartile	Crude	7.56	[1.77, 32.22]	**0.006**
		Adjusted	4.05	[0.91, 17.95]	0.066
	…above median	Crude	3.81	[2.05, 7.11]	**< 0.001**
		Adjusted	2.37	[1.17, 4.79]	**0.016**
	… in the lowest quartile	Crude	0.34	[0.17, 0.66]	**0.001**
		Adjusted	0.66	[0.32, 1.39]	0.279
PDFFquadratus	… in the upper quartile	Crude	7.84	[1.84, 33.38]	**0.005**
		Adjusted	5.12	[1.17, 22.34]	**0.030**
	…above median	Crude	2.63	[1.48, 4.67]	**0.001**
		Adjusted	1.51	[0.79, 2.87]	0.214
	… in the lowest quartile	Crude	0.15	[0.07, 0.32]	**< 0.001**
		Adjusted	0.21	[0.1, 0.48]	**< 0.001**

Results from logistic regression models with outcome IVDD. Predictors were the combination of sex-specific quartiles of PDFF_authochtonous_ and BMAT-FF_L1/L2_ (upper rows) or PDFF_quadratus_ and BMAT-FF_L1/L2_ (lower row). Crude: no adjustment, Adjusted: adjusted for age, sex, BMI, and physical activity. *BMAT-FF* bone marrow adipose tissue fat-fraction, *PDFF* proton-density fat fraction

## Discussion

In this population-based study, quantitative MRI techniques were used to assess the correlation between paraspinal myosteatosis and vertebral bone marrow composition and their associations with IVDD. We found a significant correlation between paraspinal myosteatosis and vertebral bone marrow fatty infiltration. However, both paraspinal PDFF and vertebral BMAT-FF were positively associated with the presence of IVDD and our results suggest that increased fatty infiltration of paraspinal muscle and lumbar bone marrow may be both independent as well as additive risk factors of IVDD. Although we were not able to prove causality, these findings underline the complex biomechanics of muscle, bone, and intervertebral disc that play a role in spinal degeneration.

The paraspinal muscles are spatially and functionally connected to the vertebral column and are of major importance for spine stability [29]. Despite this close relationship, only two studies assessed the association between vertebral bone marrow and paraspinal muscle composition using quantitative MRI [30, 31]. While Burian et al found no significant association between bone marrow and muscle fat composition at the lumbosacral junction, Sollmann et al reported a significant correlation of paraspinal muscle PDFF and vertebral body PDFF in postmenopausal women in a small female-only cohort [30, 31]. In contrast, no significant correlation between muscle and bone marrow PDFF was observed in premenopausal women [30]. Here, we found a significant correlation between paraspinal fatty infiltration and bone marrow adipose tissue, measured by chemical shift encoding-based water-fat MRI. Of note, the participants of this study were overweight on average (BMI 25.0–29.9), similar to those investigated by Sollmann et al. A further parameter of interest is menopausal status. Our sample was too small to support analyses stratified for this variable since we would not be able to disentangle effects of age and menopause. In line with Sollmann et al, we therefore report results for the whole sample [30]. However, future studies with larger sample sizes should further investigate this parameter.

Current concepts suggest that fat accumulation in muscle and bone follow similar mechanisms [5]. Considering bone, adipocyte accumulation occurs in the marrow cavities. Fatty infiltration of the paraspinal muscle has two components: intra- and extramyocellular lipid, both contributing to the fat component in chemical encoding-based water-fat MRI in our study [32, 33]. In the last years, several studies assessed the association of paraspinal muscle fat composition in patients with spinal disorders, including lower back pain, osteoporosis, spinal stenosis, and radiculopathy [10, 34–37]. Significant associations of paraspinal muscle fatty degeneration with decreased bone mineral density (low bone mass/osteoporosis), measured by dual-energy X-ray absorptiometry (DXA) and quantitative computed tomography (qCT), have recently been described [36, 37]. Also, several studies found associations between osteopenia/osteoporosis and higher MRI-based fat fraction measurements [38–43]. In summary, these correlations of paraspinal myosteatosis and BMAT-FF highlight a potential (inter-)relationship between bone and muscle in the development of spinal pathologies such as osteoporosis.

Intramyocellular lipids display metabolic activity by producing proinflammatory mediators. Since it is known that pro-inflammatory mediators initiate and accelerate the process of IVDD [44], there may be a link between myosteatosis and IVDD. Numerous previous studies reported an association between paraspinal myosteatosis and IVDD [8, 9, 45–48]. In line with these findings, we also report a significant positive association between paraspinal PDFF and IVDD. However, it has to be mentioned that these studies used qualitative or semi-quantitative measurements such as the cross-sectional area (CSA) or the visual Goutallier-score for determining paraspinal fatty infiltration. It is known that semi-quantitative visual grading systems such as the Goutallier-score are prone to significant inter- and intraobserver variability [49]. Thus, a strength of our study is the use of DIXON-based quantitative MRI for myosteatosis measurements, a reliable method with good concordance to histology and validated inter- and intraobserver reliability [19, 33, 50].

In a retrospective study of 72 community-based subjects, Teichtahl et al reported a positive association between lumbar IVDD, Modic changes of the vertebral endplates, and a high paraspinal muscle fat content, suggesting that IVDD may be considered a “whole-organ” pathology affecting intervertebral discs, muscle, and the bone [8]. In line, we found a positive association of vertebral BMAT-FF with the presence of IVDD. Also, there was an increase in paraspinal PDFF and BMAT-FF according to the maximum lumbar spine Pfirrmann grade and the presence of severe disc degeneration at two or more lumbar motion segments. Moreover, our findings suggest that the associations of vertebral BMAT-FF and paraspinal PDFF with IVDD are likely independent of each other, which further supports the hypothesis of a “whole-organ” pathology. Prior to the present analysis, only two studies performed quantitative MRI to assess the association between vertebral bone marrow fat and IVDD [13, 51]. In agreement with our results, Krug et al and Ji et al found significant associations between disc degeneration adjacent vertebral body bone marrow fat [13, 51].

Our study has several limitations. First, our results are limited by relatively small sample size and the cross-sectional study design, therefore requiring confirmation in larger, longitudinal cohort studies. In addition, MR-based results of myosteatosis measurements were not compared to histopathology which is still considered the current gold standard for quantification of fat content. Sample PDFF measurements on a single axial slice at the level of the L3 vertebra, as performed in this study, may not reflect the exact distribution of lipid storage within the whole. Yet, previous studies have demonstrated the validity and reproducibility of a standardized, anatomic landmark-based quantification of skeletal muscle fat via measurement of PDFF and good concordance to histology [19, 33, 50].

In this study, we used the L1 and L2 vertebral bodies for BMAT-FF measurements. These levels may not be representative of the entire lumbar spine, as previous studies found differences in vertebral bone marrow fat fractions with an increase of BMAT-FF in the craniocaudal direction [13, 52]. Yet, considering that the lower lumbar spine (L3-L5) usually is the level of most severe degenerations and the described craniocaudal increase of BMAT-FF in lumbar vertebrae, it may be speculated that the effects reported in our study are rather conservative estimates of the association between lumbar spine BMAT-FF and IVDD. However, further studies are needed to confirm this hypothesis.

Our MRI sequence for BMAT-FF measurements did not account for T2* effects, therefore likely overestimating fat fraction when compared to other methods like MR spectroscopy [53]. Also, it did not allow sub-stratification of different lipids [54]. Despite this, our analysis focused on relative differences between groups and was not focused on providing reference values.

## Conclusion

Paravertebral PDFF and vertebral BMAT-FF were significantly correlated; however, they likely represent independent risk factors for IVDD in a cohort of the general population. Our results suggest a potential relationship between the fat composition of muscle and bone and a possible role in the process of IVDD. Therefore, this study further underlines the concept of spine degeneration as a “whole-organ” pathology. In this context, quantitative MRI measurements of paraspinal myosteatosis and vertebral bone marrow adipose tissue may serve as surrogate imaging biomarkers for individuals at risk for IVDD. However, further studies are needed to elucidate the pathophysiological relations of paraspinal muscle, vertebral bone marrow, and IVDD.

## Abbreviations and acronyms

BMAT-FF: Bone marrow adipose tissue fat-fraction
BMI: Body mass index
IVDD: Intervertebral disc degeneration
MRI: Magnetic resonance imaging
PDFF: Proton-density fat-fraction

## Declarations

### Guarantor

The scientific guarantor of this publication is Thierno D. Diallo.

### Statistics and biometry

One of the authors, Susanne Rospleszc, has significant statistical expertise.

### Study subjects or cohorts overlap

Some study subjects have been previously reported in studies based on cohort from the “Cooperative Health Research in the Region of Augsburg” (KORA) [1, 2]. Exemplary publications that included analyses of the variables: paraspinal muscle, bone marrow fat, and intervertebral disc degeneration will be listed in an additional file. None of these studies analyzed the correlation of the respective variables or their association with each other.

### References

1. Holle R, Happich M, Lowel H, Wichmann HE, Group MKS (2005) KORA--a research platform for population based health research. Gesundheitswesen 67 Suppl 1:S19-25

2. Bamberg F, Hetterich H, Rospleszcz S et al (2017) Subclinical Disease Burden as Assessed by Whole-Body MRI in Subjects With Prediabetes, Subjects With Diabetes, and Normal Control Subjects From the General Population: The KORA-MRI Study. Diabetes 66:158-169

### Methodology

• retrospective

• cross sectional study

• multicenter study
